# Experimental Techniques to Assess Coral Physiology *in situ* Under Global and Local Stressors: Current Approaches and Novel Insights

**DOI:** 10.3389/fphys.2021.656562

**Published:** 2021-06-07

**Authors:** Walter Dellisanti, Jeffery T. H. Chung, Cher F. Y. Chow, Jiajun Wu, Mark L. Wells, Leo L. Chan

**Affiliations:** ^1^State Key Laboratory of Marine Pollution, City University of Hong Kong, Kowloon, China; ^2^Department of Biomedical Sciences, City University of Hong Kong, Kowloon, China; ^3^Centre for Biological Diversity, Scottish Oceans Institute, School of Biology, University of St Andrews, St Andrews, United Kingdom; ^4^School of Marine Sciences, University of Maine, Orono, ME, United States; ^5^State Key Laboratory of Satellite Ocean Environment Dynamics, Second Institute of Oceanography, Ministry of Natural Resources, Hangzhou, China; ^6^Hong Kong Branch of Southern Marine Science and Engineering Guangdong Laboratory, Guangzhou, China

**Keywords:** environmental monitoring, underwater respirometry, fluorometry, photobiology, coral metabolism

## Abstract

Coral reefs are declining worldwide due to global changes in the marine environment. The increasing frequency of massive bleaching events in the tropics is highlighting the need to better understand the stages of coral physiological responses to extreme conditions. Moreover, like many other coastal regions, coral reef ecosystems are facing additional localized anthropogenic stressors such as nutrient loading, increased turbidity, and coastal development. Different strategies have been developed to measure the health status of a damaged reef, ranging from the resolution of individual polyps to the entire coral community, but techniques for measuring coral physiology *in situ* are not yet widely implemented. For instance, while there are many studies of the coral holobiont response in single or limited-number multiple stressor experiments, they provide only partial insights into metabolic performance under more complex and temporally and spatially variable natural conditions. Here, we discuss the current status of coral reefs and their global and local stressors in the context of experimental techniques that measure core processes in coral metabolism (respiration, photosynthesis, and biocalcification) *in situ*, and their role in indicating the health status of colonies and communities. We highlight the need to improve the capability of *in situ* studies in order to better understand the resilience and stress response of corals under multiple global and local scale stressors.

## Introduction

Coral reef ecosystems are hotspots of biodiversity and productivity in the ocean (Roberts et al., 2002) that exceed that of tropical rainforests (Ray, 1988). They provide crucial ecosystem functions and services such as providing goods for subsistence and economic fisheries, coastline protection from storms, and centers of the growing field of ecotourism (Cesar et al., 2003; Knowlton et al., 2010; Van Zanten et al., 2014). As a key habitat-forming taxa, corals are critical to both reef systems and the coastal human populations that rely on them, and it is imperative to accelerate advances to ensure the longevity and survival of corals and coral reefs.

Coral reefs have drastically declined worldwide in the last 30 years because of recruitment failures, reduced growth rates, and acute and chronic mortalities (Hughes et al., 2011, 2019), with only a fraction expected to survive in their current form over the next three decades in the Indo-Pacific region (Bruno and Selig, 2007; Descombes et al., 2015). One of the most significant and widespread anthropogenic causes of this degradation is the change in climate drivers associated with the rise in atmospheric carbon dioxide (CO_2_) and other greenhouse gases (Goulletquer et al., 2014; Hannah, 2016). Local stressors also go hand in hand with global stressors, such as coastline erosion or development, which threaten the resilience of corals through pollution and sedimentation (D’Angelo and Wiedenmann, 2014; Silbiger et al., 2018; Loiola et al., 2019; Jones et al., 2020).

**Figure 1:**
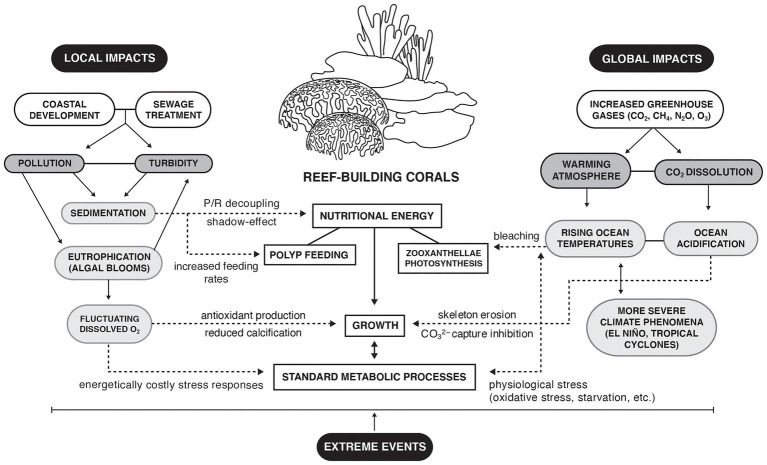
Local and global impacts affecting the nutritional energy of reef-building corals. Full lines indicate direct interactions; dotted lines indicate indirect interactions.

Increased energy consumption since the Industrial Revolution has led to the highest CO_2_ levels recorded in the atmosphere since human evolution (>410 ppm; Dlugokencky and Tans, 2020). Of the multiple greenhouse gases [such as N_2_O, CH_4_, or chlorofluorocarbons (CFCs)] affecting global biogeochemical cycles, biodiversity, and human health (Galloway et al., 2004; Bustamante et al., 2012), CO_2_ has been the most relevant to marine ecology because of its dual role in marine heatwaves (Gruber, 2011) and ocean acidification (Ciais et al., 2013; Figure 1). Overall average seawater temperatures in tropical regions have increased by almost 1°C over the past 100 years and are projected to continue increasing at 1–2°C per century (Kuffner et al., 2015). Increased seawater temperatures are a major contributor to coral bleaching and are considered as the limiting factor for coral survival (Hughes et al., 2017b, 2019). Roughly half of the CO_2_ emitted into the atmosphere dissolves into the surface ocean, reacting with water to form several dissolved inorganic components of the carbonate system (Zeebe and Wolf-Gladrow, 2001) and lowering seawater pH (Anthony et al., 2008). In comparison with pre-Industrial Revolution levels, seawater pH has decreased by approximately 0.1 (Caldeira and Wickett, 2003), which equates to roughly 30% increase in acidity and may decrease further by 0.06 to 0.32 based on emission scenarios (Ciais et al., 2013). This process of ocean acidification is particularly disruptive to marine organisms like reef-building hard corals that create calcium carbonate skeletons, increasing the energy requirements for growth and survival (Anthony et al., 2008; Cohen and Holcomb, 2009; Eyre et al., 2014). Thus, corals and coral reefs may be significantly more vulnerable than previously thought when considering the combined effects of ocean acidification and warming (Hoegh-Guldberg et al., 2007; Pandolfi et al., 2011).

Although climate drivers are widely recognized as dominating factors in coral loss and reef ecosystem shifts (Hughes et al., 2017b), localized stressors also are impacting coral health, where a survival-resilience pattern is observable in urban subtropical reefs subjected to several anthropogenic stressors (Heery et al., 2018). Here, there are indications that stress-tolerant hard coral species have been selected to foster more resistant though less diverse reefs (Darling et al., 2012). Impacts from eutrophication, increased turbidity, and lowered dissolved oxygen significantly affect coral metabolism, changing energy pathways and reef ecology (Figure 1). A coral as a holobiont includes diversity functional, genomic, and potential epigenetics traits that regulate its ecological plasticity under an environmental change. But not all coral species showed the same response patterns under anthropogenic threats.

Turbid water conditions commonly occur within inshore shallow coastal waters (Browne et al., 2014), owing to the collective interactions of river runoff and the natural re-suspension of sediments (e.g., tides or storms) as well as anthropogenic activities (e.g., ship wakes, coastline modification, and storm and other sewage discharge). The adaptive responses that corals require to survive these conditions are both stressful and energetically costly (Brown and Bythell, 2005), utilizing energy that otherwise would be put towards growth and reproduction. One consequence of increased turbidity is the reduction of the *in situ* irradiance and thereby photosynthesis, while high levels of settling sediments can hinder feeding or smother coral polyps (Fabricius, 2005). The resulting decreases in photosynthetic efficiency and increases in respiration lower the daily productivity of corals [measured as the ratio of photosynthesis to respiration (P/R)]. This decreased productivity, in turn, lowers the coral nutritional energy reserves (e.g., reduced lipids content and changes in lipids class composition), which can lead to mortality (Weber et al., 2012; Jones et al., 2020). Lower productivity also can increase coral susceptibility to infection and bleaching (Anthony et al., 2007), generating community- and ecosystem-level impacts. In contrast, corals associated with urban developments are frequently exposed to acute sedimentation but appear to escape tissue mortality by better acclimation to low light conditions (Dubinsky et al., 1984) and by increasing their feeding rates to offset energy deficits from photosynthesis (Anthony and Fabricius, 2000). However, any increased frequency or severity of acute sedimentation contributes additional stress to corals that can be functioning near the limits of their physiological tolerances (Bessell-Browne et al., 2017; Loiola et al., 2019; Jones et al., 2020).

Although increased planktonic production can enhance the food supply for corals, very high photosynthetic production rates can generate hyperoxic conditions in reef waters that can cause coral damage and photorespiration (Mass et al., 2010). At best, hyperoxic conditions may shift coral energy use into the production of antioxidants rather than calcification (Wijgerde et al., 2014). In contrast, low oxygen concentrations also challenges coral survival, stimulating metabolic acclimation through the expression of hypoxia-inducible factors in corals (Alderdice et al., 2021). There are few reports of hypoxic events and dead zones in the tropical coral reef environments, though episodic or seasonal hypoxia has been recently linked to bleaching and mortality in deeper water corals (Altieri et al., 2017).

The anthropogenically derived increased inputs of nutrients and organic matter in coastal regions have degraded many coral reefs, and this cultural eutrophication might exacerbate the effect of global warming on coral survival (Silbiger et al., 2018; DeCarlo et al., 2020). The nutrient gradient affects the nutritional status of corals, in particular when the calcification rate is reduced but heterotrophy enhanced under high nutrient loading (Sawall et al., 2011). Indeed, traces of nitrate pollution can be found in both hard and soft corals through the analysis of radioisotopes δ^15^N in the tissue (Baker et al., 2011; Duprey et al., 2017) and δ^13^C as an estimate of particulate organic matter ingestion (Baker et al., 2010; Conti-Jerpe et al., 2020). At the community level, a shift from net community calcification (NCC) to dissolution can occur under high nutrient conditions (Silbiger et al., 2018), due to combination of direct and indirect responses of corals. Indeed, nutrient enrichment might negatively affect the physiological performance of coral metabolism, increase the productivity of reef macroalgae, or both, inducing a cascade of change in the coral ecosystems (D’Angelo and Wiedenmann, 2014; Silbiger et al., 2018). Eutrophic conditions can increase the productivity of reef waters by increasing food availability (e.g., particulate matter; Fabricius, 2005), although macroalgae, turf, and bioeroders can inhibit competitively the coral recruitment (D’Angelo and Wiedenmann, 2014). However, several factors influencing the susceptibility to eutrophication have to be included, such as hydrodynamic connectivity and location (Fabricius, 2011). Even so, Sawall et al. (2011) found higher photosynthetic rates of coral endosymbionts nearshore along an anthropogenic driving inshore-offshore nutrient gradient, showing that eutrophication effects on coral may not always be negative.

Gaining knowledge of the stress responses of corals and their effects on reef ecology, along with the pathways to best minimize these impacts, depends on two related tasks: understanding coral health from polyp-endosymbiont symbiosis to the community level and achieving early detection of the onset of the stress responses. The first provides the foundation for studying and developing potential mitigation and managements strategies, and the second is crucial for implementing these strategies soon enough to help minimize impacts.

While most early studies of an environmental change on coral health focused on the effects of single drivers (e.g., temperature, ocean acidification, and turbidity), their interactive effects require addressing multiple stressor effects on physiological processes at the holobiont level if we are to comprehensively understand their impacts on coral communities. Some of these drivers have major effects (e.g., temperature and light), but there undoubtably are other interactions that can affect the resistance and recovery response of coral communities, factors critical for reef management and proactive preventative planning. However, our current understanding of coral stress responses is largely based on experimental manipulation studies in laboratory systems that are poor representations of their natural habitats. *In situ* studies, by either SCUBA or automated sensors, could provide a better understanding of local and global impacts, but only a few recent studies have undertaken the logistical complexities of studying fine-scale physiological processes of corals *in situ* recently (Roth et al., 2019; Cyronak et al., 2020; Srednick et al., 2020). The primary objective of this review is to summarize the current strategies for quantifying aspects of coral metabolism to highlight the benefits of non-destructive methodologies. A list of recommendations is provided that would expand the efficacy of underwater studies for improving local knowledge and better understanding of how corals respond to stressors.

## Overview of the Coral Metabolism

### Metabolic Responses at Coral Polyp Level

The concept of the coral as a “holobiont” was introduced in the 2000s, whereby the coral comprises not only the animal polyps but also the associated symbiotic organisms, including photosynthetic endosymbionts, bacteria, viruses, fungi, and protists (Rohwer et al., 2002; Rosenberg et al., 2007). These have a fundamental role in nutrient and energy acquisition processes of coral polyps (Peixoto et al., 2017), genome evolution in the coral host and microbial partners, maintaining holobiont homeostasis, and the overall health of the holobiont (Thompson et al., 2015). The functional diversity of these microbes contributes to the stress response and acclimation to changing conditions, e.g., high seawater temperature and bleaching events (Jones et al., 2008; Hume et al., 2016).

The family Symbiodiniaceae includes several genera of dinoflagellates, which reside in the tissue of corals and other marine organisms (LaJeunesse et al., 2018). The coral-Symbiodiniaceae association is an obligate symbiosis for the coral, where the photosynthetic dinoflagellates provide the coral oxygen and energy in the form of glucose for aerobic respiration (Muscatine, 1998; Gardella and Edmunds, 1999). While most of the oxygen arising from photosynthesis is immediately utilized in coral respiration (Kuhl et al., 1995; Al-Horani et al., 2003a,b), the excess oxygen is released to the surrounding seawater throughout the day (Yonge et al., 1932; Finelli et al., 2006; Al-Horani et al., 2007; Mass et al., 2010), supporting the oxygen availability on the reef. Therefore, at night, corals must acquire oxygen from the surrounding environment to fuel the respiration process (Al-Horani et al., 2007). As a consequence, the energy acquisition follows a circadian pattern where endosymbiont-derived photosynthesis is dominant during the day while polyp-guided respiration is dominant at night.

This continuous loop is stable under normal conditions, but the symbiotic dynamics within the coral holobiont are changing rapidly in the Anthropocene (Hughes et al., 2017a). Temperature-induced stress damages a key protein (D1) in the dinoflagellate photosystem II within the dinoflagellate (PSII; Hill et al., 2011), inactivating the Rubisco center (Lilley et al., 2010), affecting the production of ATP (Franklin et al., 2004), and leading to the overproduction of reactive oxygen species that can induce damaging conditions for both the endosymbiont and coral host. The increased frequency and severity of temperature anomalies will lead to negative consequences for coral survival (Anthony et al., 2008; Randall and Szmant, 2009). Under warming and eutrophic conditions, coral host-endosymbiont relationship may shift from a mutualistic to parasitic strategy with respect to nutritional resource allocation (Lesser et al., 2013). This shift has important implications for the resilience of coral reefs under bleaching conditions, where decreases in net primary productivity of the holobiont mainly from increased respiration in the host, with little apparent metabolic cost to the endosymbiont (Douglas, 2008; Baker et al., 2018).

The coral symbioses also can be influenced by other types of stress related to environmental changes. The impact of high seawater pCO_2_/low pH on corals is highly variable, where it can stimulate growth and photosynthetic efficiency in certain Symbiodiniaceae species (Brading et al., 2011). In other cases, increasing pCO_2_ had no measurable effect on net photosynthesis (gross photosynthesis – respiration) but decreased biocalcification rates (Comeau et al., 2017). Similarly, elevated concentration of nitrates (NO_3_^−^) or phosphate (PO_4_^3−^) can stimulate both photosynthesis and respiration rates in corals, but not necessarily result in a concomitant increase in net biocalcification (Silbiger et al., 2018). Such imbalance between photosynthetic energy supply and biocalcification (as biologically induced formation of CaCO_3_) may indicate increasing competition for dissolved inorganic carbon (DIC) between coral host and endosymbionts under conditions of high production. Adding further complexity to these responses, ammonia (NH_4_^+^) enrichment appears to support coral resistance to thermal stress, unlike enrichments of NO_3_^−^, which can lead to increased levels of reactive species and oxidative damage (Fernandes de Barros Marangoni et al., 2020).

Bleaching, where the endosymbionts may decrease their chlorophyll content or be ejected from the coral host, signals severe physiological consequences arising from oxidative stress and starvation, leading to reduced respiration rates and energy acquisition (Williams et al., 2017). Though the role of endosymbiont reactive oxygen species production is believed to be fundamental to this process, recent work shows that coral bleaching is more complex than previously thought (Rädecker et al., 2021). Although bleaching can be a reversible condition, it often leads to coral tissue death if sustained too long. However, endosymbionts have developed adaptive mechanisms to limit thermal stress by increasing electron flow in photosystem I as photoprotection (Hoogenboom et al., 2012), thereby limiting the extent of bleaching. Moreover, coral polyps may partially compensate for the lost photosynthetically derived energy during bleaching by enhanced feeding if sufficient prey are available (Anthony et al., 2007).

### Responses of the Coral Community

When magnified across coral tissues, the polyp-level metabolic responses can affect reefs at the community level. Growth of coral colony, or NCC, is measured in terms of the deposition of its calcium carbonate (CaCO_3_) foundation, which is determined largely by coral energy reserves and the need to maintain optimal chemical conditions in the calcifying fluid at the tissue/calcium carbonate interface. Hard corals build their skeleton of CaCO_3_ through the uptake of calcium and carbonate ions from seawater. The reaction occurs in the calicoblastic cells lining the surface where primary crystal secretion occurs (Cohen and Holcomb, 2009) and is facilitated at pH levels above that of seawater. However, ocean acidification reduces the seawater pH and the aragonite saturation state (a; Eyre et al., 2014), which can have a strong effect on NCC, along with the balance of organic matter production and respiration, or net community production (NCP; Albright et al., 2015). Moreover, the projected decreases in pH in the future ocean eventually will affect the stability of existing reef ecosystems, because the CaCO_3_ formed by corals (aragonite) is more susceptible to dissolution than that formed by other biocalcifying organisms (Shaw et al., 2013). These effects overlay natural seasonal variations in the carbonate chemistry in reef systems, and the question remains of what extent does globally and locally derived ocean acidification have on the seasonal balance of NCP and NCC (Cyronak et al., 2018). However, these estimates remain imprecise, owing to the lack of information about seawater residence time and volume, which would modulate reef chemistry (Courtney et al., 2017). The relationship between host and endosymbionts is exacerbated under the combined effects of global drivers of stress (e.g., temperature, ocean acidification, and atmospheric conditions), leading to lower energy reserves for growth (Anthony et al., 2008; Hughes et al., 2011, 2018; Hoegh-Guldberg, 2014). These, in turn, are combined with local drivers in coastal regions (e.g., increased nutrient, turbidity, and freshwater inputs), causing reduced calcification and oxidative stress and eventually leading to bleaching events (Silbiger et al., 2018; Fernandes de Barros Marangoni et al., 2020).

Even with these impacts though, it is possible for coral communities to recover after destructive events. For example, the coral reef in Kane’ohe Bay showed positive NCC and NCP about 1 year after the last drastic bleaching event in 2015 (Courtney et al., 2018), indicating the capacity of local rapid post-bleaching recovery. However, these estimates considered only the abiotic factors, such as carbonate chemistry and oceanographic patterns, without considering potential effects from the benthic community, which would contribute to the depletion or repletion of alkalinity in the coral reef (Page et al., 2017).

Defining the health of corals and coral communities is complex given the array of elements that contribute the status of reef ecosystems. The coral microenvironment comprises ecologically dependent niches (a network made up of bacteria, viruses, and fungi) that supports and regulate the general homeostasis and plasticity of the coral holobiont. But understanding these *in situ* responses to variable multistressor conditions cannot be adequately informed by just laboratory-based manipulation studies, so the adaptive resilience of coral species and their response thresholds remain only partially understood. It is difficult therefore to chart ways forward for pragmatic and effective steps towards protection and restoration. Overcoming these limitations will depend on applying new *in situ* observational techniques that can bridge from single organisms to ecosystems and on to regional global scales.

## Materials and Methods

We conducted a literature review based on case studies of *in situ* observations of coral metabolism from published peer-reviewed scientific literature. We included cases involved in original research on direct (e.g., using SCUBA divers, benthic chambers, and optical sensors) underwater measurements of metabolic rates on both coral individuals and coral communities. We excluded sample collections (e.g., coral fragmentation), laboratory experiments, and indirect measurements of metabolic fluxes (e.g., *ex situ* from sample incubations).

We used the advanced search on Google Scholar to identify studies with the keywords “coral *in situ* metabolism” and “underwater,” excluding the keyword “collection,” in articles published between 1991 and 2020. The search yield (*n* = 2,090) was scrutinized, and the literature was manually reviewed to fulfill our selection criteria described above. A final list of 55 studies was included for the analysis in this review (Table 1).

**Table 1:** Reference studies discussed in this review, listed in chronological order of publication.

ID no.	Reference
1	Patterson et al., 1991
2	Gattuso et al., 1993
3	Beer et al., 1998
4	Ralph et al., 1999
5	Gorbunov et al., 2000
6	Lombardi et al., 2000
7	Lesser and Gorbunov, 2001
8	Banaszak et al., 2003
9	Winters et al., 2003
10	Yates and Halley, 2003
11	Levy et al., 2004
12	Okamoto et al., 2005
13	Wild et al., 2005
14	Levy et al., 2006
15	Carpenter and Patterson, 2007
16	Finelli et al., 2007
17	Nakamura and Nakamori, 2007
18	Weber et al., 2007
19	Frade et al., 2008
20	Piniak and Storlazzi, 2008
21	Nakamura and Nakamori, 2009
22	Schneider et al., 2009
23	Carpenter et al., 2010
24	Sawall et al., 2011
25	McGillis et al., 2011
26	Mass et al., 2011
27	Kline et al., 2012
28	Suggett et al., 2012
29	Wangpraseurt et al., 2012b
30	Haas et al., 2013
31	Ferrier-Pagès et al., 2013
32	Okazaki et al., 2013
33	Roder et al., 2013
34	Long et al., 2013
35	Wangpraseurt et al., 2014
36	Shaw et al., 2014
37	Treibitz et al., 2015
38	Rovelli et al., 2015
39	Camp et al., 2015
40	Takeshita et al., 2016
41	Chow et al., 2016
42	Biscéré et al., 2017
43	Albright et al., 2018
44	Takeshita et al., 2018
45	McMahon et al., 2018
46	Roth et al., 2019
47	Cyronak et al., 2020
48	Ramesh et al., 2019
49	de Froe et al., 2019
50	Doo et al., 2019
51	Pramneechote et al., 2020
52	Srednick et al., 2020
53	Oh et al., 2020
54	Roth et al., 2020
55	Dellisanti et al., 2020

**Figure 2:**
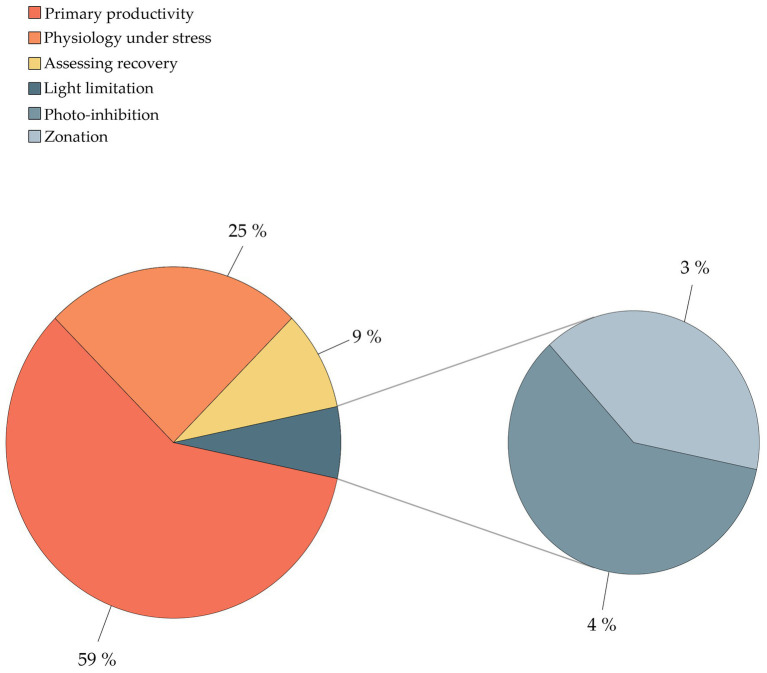
Study objectives of *in situ* methodologies for the measurement of coral physiology.

**Figure 3:**
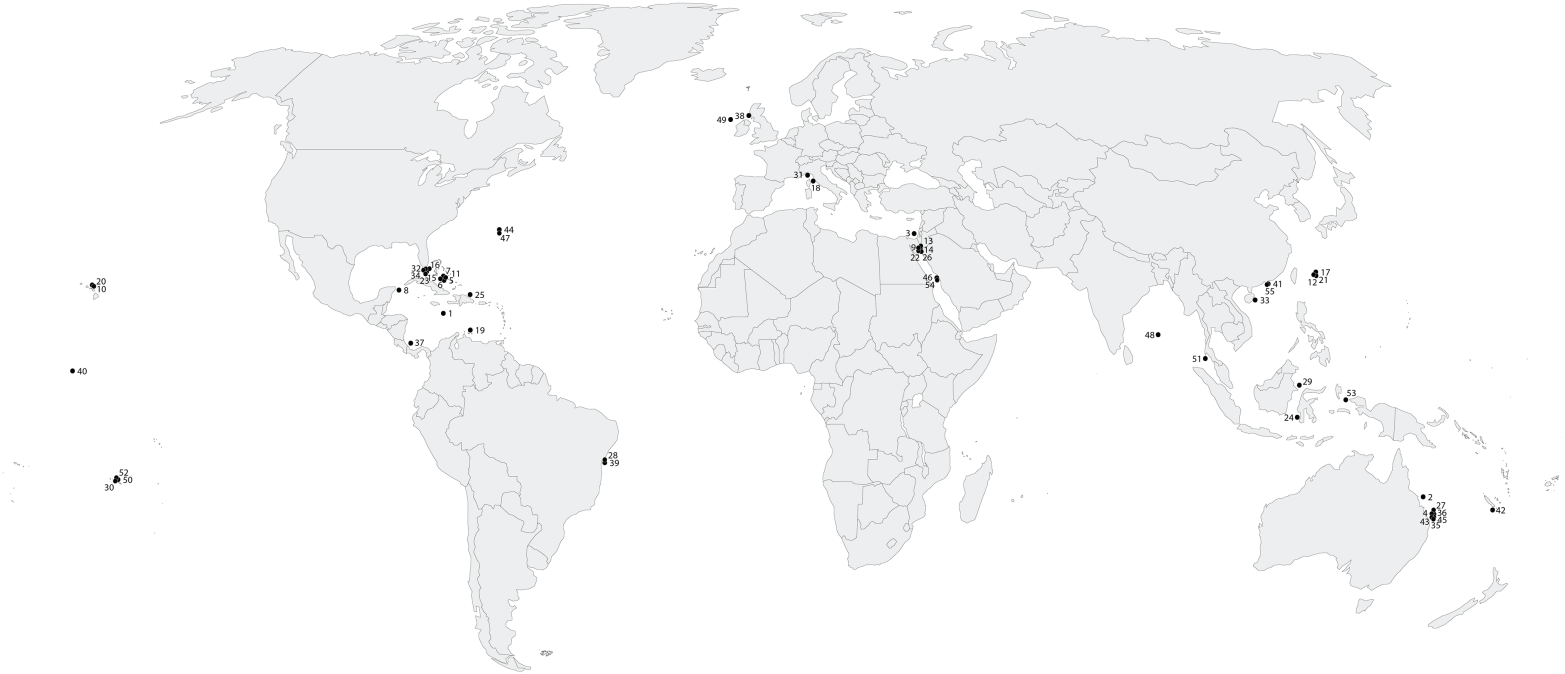
Global map of scientific studies cited in this review. The reference identification numbers are listed in Table 1.

The literature screening was categorized in eight separate categories based on the methodology used (Table 2), including (1) SCUBA fast repetition rate (FRR) fluorometry; (2) SCUBA pulse amplitude modulated (PAM) fluorometry; (3) Clark-type O_2_ sensors; (4) boundary layer; (5) SCUBA imaging; (6) benthic chambers; (7) submersible chambers; and (8) automated sensors. The database was further classified based on the system type (open, semi-closed, and enclosed); measured parameters [O_2_, photosynthetic efficiency (Fv/Fm), electron transport rate (ETR), and calcification rate (CA)]; sampling frequency (minutes, hours, and days); sampling scale (symbionts, polyp, colony, and community); aim of the study (monitoring and experiment); and environmental stressor considered (light, temperature, turbidity, water quality, water flow, ocean acidification, and time). We ranked the database by publication year (Table 3) and identified the most commonly used techniques for studying *in situ* coral metabolism. We analyzed the objectives of the studies included in this review (Figure 2) in order to give an overview on the topics mostly studied between 1991 and 2020. Finally, we collated all 55 studies into a map to show the global distribution of experimental studies *in situ* discussed in this review (Figure 3).

**Table 2:** Experimental techniques available for *in situ* monitoring of coral physiological processes.

Method	System	Parameters	Sampling frequency	Sampling scale	Aim	Stressor	Reference ID no.
SCUBA FRR fluorometry	Open	ETR, Fv/Fm	Minutes	Symbionts	Monitoring	Light	5–7, 11, 14, 37
SCUBA PAM fluorometry	Semi-closed	ETR, Fv/Fm	Minutes	Symbionts	Monitoring	Light, temperature, water flow, water quality	3, 8, 9, 12, 15, 16, 19, 20, 24, 28, 33
Clark-type O_2_ microsensor	Open	O_2_	Hours	Symbionts, polyp	Experiment, monitoring	Water quality	18, 49
Diffusive boundary layer	Semi-closed	O_2_, CA	Minutes	Symbionts, polyp	Monitoring	Light, water quality, time	10, 25, 34, 35, 38, 40
SCUBA imaging	Open	O_2_	Hours	Symbionts, polyp, colony	Monitoring	Light, water quality	41, 48, 53
Benthic chamber	Enclosed	O_2_, CA	Days	Colony	Experiment, monitoring	Light, water flow, water quality, ocean acidification	1, 2, 13, 21–23, 26, 30–32, 39, 42, 46, 52–55
Submersible chamber	Enclosed	O_2_, CA	Hours	Colony, community	Experiment, monitoring	Light, water quality, ocean acidification	11, 14, 21, 22, 26, 39, 43, 50, 51
Automated sensors	Open	O_2_, CA	Hours	Community	Monitoring	Water quality, ocean acidification	40, 43–45, 47

SCUBA FRR, SCUBA-based fast respiration rate fluorometer; SCUBA PAM fluorometry, SCUBA-based pulse amplitude modulation; O_2_, dissolved oxygen; CA, calcification; ETR, electron transport rate; Fv/Fm, maximum quantum yield of photosystem II; Stressors, includes environmental stressors considered in the study; Reference, the identification number is referred to Table 1.

**Table 3:** Time ranking of published peer-reviewed studies on coral *in situ* metabolism according to this study.

Year	Methodology	Objective	Sampling scale
B41991–2005	Fluorometry, benthic, and submersible chamber	Productivity, diel change	Polyp, colony
2006–2010	Fluorometry, benthic, chamber and automated sensors	Diel change, productivity, physiology under stress	Colony, community
2011–2015	Benthic and submersible chamber, diffusive boundary layer	Physiology under stress, productivity	Colony, polyp, community
2016–2020	Benthic and submersible chamber, automated sensors	Seasonal change, productivity, physiology under stress	Colony, community

## Results

### Methodologies and Objectives for Coral *in situ* Metabolism

Several tools and instruments have been developed to noninvasively measure coral metabolic and physiological processes in both field and laboratory studies. These methods can be used to distinguish among healthy and stressed organisms, and the transitions between these states. We focus here on diver-portable technologies, designed to non-destructively estimate energy production and expenditure, such as respirometers and fluorometers. Although there is little consensus about the extent to which apparently healthy corals are adapting to the changing conditions (Baker et al., 2008), measures of metabolic proxies (e.g., variations in dissolved oxygen and pH) have been used at both individual coral and coral community scales to infer the metabolic state of benthic ecosystems (Bates et al., 2010). Quantitative links then are sought between these coral indicators and the wider status of the ecosystem physical and biogeochemical indicators.

Laboratory-based studies of coral metabolism in almost all cases involve the destructive sampling of individual colonies or fragments thereof. Although this framework provides crucial knowledge on isolated physiological responses, mechanisms, and pathways under well-controlled conditions, the study conditions cannot comprehensively reflect the stochasticity and cyclical fluctuations in environmental conditions on a reef, nor the cumulative effects of multiple chronic or acute stressors. In the last decades, experimental techniques to study the metabolic status of corals directly *in situ* have been developed, using specific tools engineered for automated analysis on coral surface and reef communities. Among these, microsensors and benthic chambers for respirometry and fluorometry techniques became the most popular for coral studies over a wide range of topics (Table 2).

The focus of instrumentation development for the *in situ* study of coral metabolism has shifted over the last three decades, from using fluorometric techniques to estimate endosymbiont photosynthetic efficiency to a more comprehensive investigation of the holobiont relationships in individuals and communities (Table 3). Indeed, through the use of benthic chambers, it is possible to run experimental manipulations of carbon or nutrient fluxes and monitor long-term ecosystem responses *via* automated sensors. With this shift, the linkages among biogeochemical and physiological processes in corals have greatly improved the understanding of coral ecosystems functioning to where modeling now has a realistic possibility to predict benthic conditions under future climate scenarios (Roth et al., 2019).

Among the objectives of coral physiology studies, 59% of the published works comprised investigations of the diel or seasonal changes in primary productivity, highlighting that quantification of natural variations in energetic fluxes is a central concern for the assessment of coral conditions (Figure 2). Moreover, coral primary productivity was the only objective of these studies being covered by all methodologies at different sampling scales, from polyp to community. Conversely, 25% of studies addressed coral physiology under stress, including bleaching responses, environmental changes, and bathymetric change conditions (transplantation). The assessment of recovery of health status was investigated by 9% of the studies. The remaining papers centered on issues of light limitation, including zonation (4%) and photo-inhibition (3%).

### Underwater Fluorometry

The photochemical performance of coral endosymbionts is a key aspect influencing the health of scleractinian corals, so measures of holobiont photophysiology provide a valuable insight into coral metabolic status. Fluorometry can provide this insight in two broad ways: (i) by characterizing the abundance, character, and distribution of light-harvesting pigments (potential energy source) and fluorescent proteins (a sign of immune response and tissue repair) in corals and (ii) as an indicator of how efficiently these pigments are being used to generate energy for the coral, indicated by the photo-physiological status of the endosymbiont.

A fundamental measure of coral status is quantification of the light-harvesting pigments of the endosymbionts over time, whereby decreasing trends may provide early warning of the onset of bleaching events. The underwater fluorescence imaging has been adapted to conduct large-scale *in situ* assessments of coral reefs, through the detection of chlorophyll and green fluorescent proteins wide spatial scales (Treibitz et al., 2015; Oh et al., 2020). Chlorophyll concentrations are indicative of photo-acclimation, among other things, and green fluorescent proteins may, in addition to being a signal of tissue repair (D’Angelo et al., 2012), contribute to photoprotection in corals; and thus, quantifying these concentrations can provide crucial information on the bleaching susceptibility or resistance of corals (Salih et al., 2000; Kenkel et al., 2011; Roth and Deheyn, 2013). In addition, this imaging technique can also mark early signs of disease as well as help to detect coral juveniles thereby leading to more accurate assessment of coral reef health (Treibitz et al., 2015; Ramesh et al., 2019).

Early *in situ* studies on coral physiology estimated the rates of photosynthesis and respiration within recirculating chambers by measuring changes in oxygen concentrations (Patterson et al., 1991). While still a highly useful approach (see below), the introduction of variable fluorescence techniques has greatly increased the ability to assess the photophysiology of the endosymbionts and thus provide a sensitive indicator of their metabolic status. With variable fluorescence, very rapid pulses of light are used to probe the photosystems and inform on a number of photosynthetic parameters including the quantum yield of photosystem II (PSII), which is used as a metric for photosynthetic efficiency (Schreiber et al., 1986) and is a key indicator of the physiological status of the endosymbiont. Changes in photosynthetic efficiency provide an early signal of stress, some of which are natural responses to changes in irradiance but become more prolonged and severe when conditions of imminent bleaching occur.

The estimation of the potential quantum yield of PSII as a metric for photosynthetic rates was first applied to underwater organisms by Beer et al (Beer et al., 1998). PAM fluorometer uses light pulses of relatively long duration (300–1,200 ms, 3,000–10,000 mmol photons m^−2^ s^−1^) to modify the fluorescence yields, generating multiple turnover for the reduction of PSII, allowing noninvasive chlorophyll fluorescence analysis, quantum yield, and ETR of coral endosymbionts. The introduction of an underwater version of this instrument (Diving-PAM, Walz) facilitated the direct observation of changes in coral health *in situ* conditions (Ralph et al., 1999). The introduction of *in situ* PAM fluorometry enabled the construction of photosynthesis-irradiance models for corals based on varying sun exposures in reefs (Beer et al., 1998; Ralph et al., 1999). For example, observations of decreased quantum yield during the day reflected photoinhibition through loss of photosynthetic capacity, a response seen in shallow but not deeper water corals (Winters et al., 2003). Similar reduced photosynthetic efficiency has been observed in corals exposed to seasonal turbid conditions (Piniak and Storlazzi, 2008). The use of Diving-PAM continuous monitoring during bleaching events also showed that combined thermal and light stress generated greater reductions in photosynthetic efficiency and was a sensitive indicator of metabolic stress in impacted areas (Roder et al., 2013). Even so, variable responses in the coral community to these stresses suggested coral species-specific physiological responses (Banaszak et al., 2003; Okamoto et al., 2005; Frade et al., 2008; Suggett et al., 2012). Further development on PAM technology is the high-resolution imaging fluorometry used for analysis of heterogeneity within colonies, from coenosarc tissue to polyp tentacles, but so far, this methodology is not available for underwater studies (Ralph et al., 2005).

A second approach for measuring variable fluorescence is the FRR fluorometer, which uses a different approach to saturate the photochemistry during measurement. Here, shorter (150–400 ms, >20,000 mmol photons m^−2^ s^−1^) but more intense light is used to generate a single turnover and reduction of the primary electron acceptor (Kromkamp and Forster, 2003; Suggett et al., 2003). The methodology is more sensitive than PAM fluorometry, which can be beneficial when endosymbiont abundance (or chlorophyll concentrations) is very low in corals. The SCUBA-based FRR fluorometry was developed by Gorbunov et al (Gorbunov et al., 2000) for measuring chlorophyll fluorescence from PSII reaction centers in corals, sea grasses, macroalgae, and algal turfs. FRR fluorometry has been also used to monitor coral physiology during bleaching events and their later recovery (Lombardi et al., 2000; Lesser and Gorbunov, 2001). Although nutrient availability (as nitrogen or iron) and sunlight irradiance were already recognized as the main factors directly affecting photosynthesis in the aquatic ecosystem (Falkowski and Kolber, 1995; Behrenfeld and Kolber, 1999), the use of FRR fluorometry enabled more direct measurements of nutrient limitation effects on photosynthetic efficiency of corals. Indeed, the FRR fluorometry predicted which corals might be more susceptible to bleaching under stress conditions even when no signs of stress are visible, but photosynthetic quantum yield was reduced (Lesser and Gorbunov, 2001).

Submersible chambers have been used to study the evolution of diurnal hysteresis in coral photosynthesis *in situ*, enabling the comparison of respirometry and fluorometry-based techniques (see below; Levy et al., 2003, 2006). Marked discrepancies were observed, reflecting the importance of different scales of measurements. FRR and PAM fluorometry methods yield signals at the polyp-symbiont scale (Gorbunov et al., 2000), while changes in oxygen concentrations in chambers integrate the whole colony response (Schneider et al., 2009). The assumption here is that the chamber system designs and durations impart no added stress condition on the corals, which appears to be the case in many studies (Camp et al., 2015; Roth et al., 2019; Dellisanti et al., 2020). While it can be argued that chamber methods may be more informative in terms of the holobiont, the portability, ease of operation, and comparatively low cost of fluorescence instruments (Diving-PAM, FRR, and imaging systems) have greatly expanded their use for studying photosynthetic performance of coral symbionts and monitoring diel changes of coral productivity.

### Benthic Chambers, Diffusive Flux Methods, and Flow Analysis

Early *in situ* studies on coral biology used the strategy of encapsulating single or multiple individuals within closed benthic systems (Patterson et al., 1991). These chamber methods enabled the non-destructive measurement of coral physiological rates through changes in water chemistry to better understand community level responses to perturbation (Patterson et al., 1991; Gattuso et al., 1993). For example, incubation chambers have been used to study *in situ* coral calcification and photosynthesis rates and showed that ocean acidification can lower the biocalcification rates (Okazaki et al., 2013). Larger incubation systems have enabled measurements of benthic community metabolism as well as conducting *in situ* experiments, providing a better insight into ecological responses in reefs than can be the focus of studies on host-symbiont relationships (Yates and Halley, 2003; Haas et al., 2013).

The common metrics to assess the health of corals are the levels of photosynthesis by the endosymbionts, respiration of the holobiont, and ideally the rates of biocalcification (or growth). Benthic chambers are well-suited to measure these essential parameters *in situ* (Wild et al., 2005; Nakamura and Nakamori, 2007; Ferrier-Pagès et al., 2013; Camp et al., 2015). For example, differences in coral metabolism were measured between shallow (reef flat) and deeper area (reef slope) at Ishigaki Island, Japan, with negative net calcification rates being attributed to nighttime decreases in pH due to benthic respiration (Nakamura and Nakamori, 2009). In another study, Sawall et al. (2011) used benthic chamber to study the coral nutritional status at elevated anthropogenic nutrient loading. The novel incorporation of a transparent 3-L urine bag into the “Flexi-Chamber” design led to an easily managed, low-cost device for use by any underwater scientists, where short incubations are able to detect changes in biological processes absent of any visual signs of coral stress or change in endosymbionts concentration (Camp et al., 2015). A similar benthic chamber design was used to show that abiotic conditions, including light intensity, were drivers of spatial-temporal patterns of reef metabolism, although some limitations on the use of these incubation chambers emerged when applied on large boulder colonies (Pramneechote et al., 2020). Here, the amount of light available for photosynthesis and the energetic budget can vary based on colony shape (micro niches) and the immediate surrounding environment (backscattering light; Wangpraseurt et al., 2014). However, newer designs of noninvasive diver-portable *in situ* incubation chambers (~70 L, 0.2-m^2^ area) have been developed to measure biogeochemical processes in both simple (sediments) and structurally complex (corals or rocky bottoms) reef communities, allowing the *in situ* study of diverse habitats, such as corals, sediments, and seagrass meadows (Roth et al., 2019). The introduction of benthic and submersible chambers has made it possible to link individual metabolic host-symbiont processes to community-wide reef responses, improving the generality and robustness of previous metabolic and physiology studies.

An ambitious program began in mid-1996 to adapt benthic chambers for use as flow respiratory systems to study the *in situ* responses of coral communities. Using a combination of *in situ* flow chambers and fluorometric techniques, Shepard et al. (1996) demonstrated how both water flow and temperature combined to influence coral physiology. Later, the “Submersible Habitat for Analyzing Reef Quality” was designed to maintain and measure a turbulent flow of water over benthic substrates for extended periods while measuring temperature, oxygen, salinity, pH, and irradiance (photosynthetically active radiation) and to monitor the daily variations in photosynthesis, respiration, and calcification (Yates and Halley, 2003). This system enabled some of the first studies of the direct influence of high CO_2_ levels on coral communities in short-term experiments (Yates and Halley, 2003). But the real strength of this approach is the ability to study the ecology and biogeochemistry of coral/benthic communities and how they may acclimate to altered conditions over multiple days. A similar automated closed chamber was used to study coral responses over a variety of substrate types (Gevaert et al., 2011).

These flow systems have enabled new strategies for *in situ* investigation of the effects of environmental stressors on reef metabolism, ecology, and biogeochemistry. Flow systems have been used to demonstrate asymmetric patterns of photosynthetic yield across coral colonies (Carpenter et al., 2010), where the upper side has a reduced quantum yield (Carpenter and Patterson, 2007). Other experimental studies have used flow chambers to study coral physiology under chemical enrichment in a benthic chamber experiment (Biscéré et al., 2017), adding new findings on coral responses to high pCO_2_ and the combined effect of chemical inputs and global warming. Haas et al. (2013) used large collapsible benthic isolation tents (cBITs) to study the integrated responses of corals, macroalgae, and microbes over diurnal cycles, and they showed how the release of algal exudates influenced microbial metabolism and energy transfer to higher trophic levels. Similarly, small benthic chambers enclosing ~70 L (Roth et al., 2019) were used to study complex benthic structures and to measure coral reef community budgets of primary production over coral-dominated or algae-dominated reef communities (Roth et al., 2020), adding new findings on the biogeochemical cycles of coral reef ecosystems. A common feature of these flow systems is that they commonly require constructing or placement of large or cumbersome structures on the reef, so replicating findings across and among reef systems is difficult. A new diver-portable respirometer [community *in situ* metabolism (CISME)] enables short-term (minutes) quantification of coral photosynthetic, respiration, and biocalcification rates combined with the ability to study the effects of flow and chemical manipulations (Dellisanti et al., 2020). Although its use has been limited to date, it offers many of the benefits of larger chamber devices but also the ability to assess individual coral colonies across and among reef environments with good accuracy and resolution.

### Micro- and Automated Sensors

While benthic chambers provide a means for studying the community level responses, by design, these systems alter water flow dynamics around the study corals and restrict water exchange sufficiently to enable changes in water chemistry to accumulate (i.e., the measurement signals). It is unclear at what stage these changes in water flow and bulk chemical conditions may begin influencing coral responses. The use of micro- and automated sensors for unenclosed measurements has been developed to help avoid these potential artifacts. These approaches are broadly separated into community-scale and individual coral or polyp scales.

Unenclosed, diffusive boundary layer (DBL) approaches were developed to avoid these potential issues, whereby sensors placed well above the coral interface make unobstructed measurements of vertical gradients in velocity, temperature, and chemical constituents on the open reef to calculate the flux of momentum, heat, and O_2_ in the boundary layer (McGillis et al., 2011). Use of these unenclosed strategies has revealed new insights into coral metabolism by showing the fluctuations in biologically mediated changes in chemical conditions under different natural regimes of dissolved oxygen, pH, and light intensity (Cyronak et al., 2018; McMahon et al., 2018; Takeshita et al., 2018).

The use of microsensors at coral tissue levels in turn has illustrated how these factors influence and are influenced by the coral-seawater interface (McGillis et al., 2011; Wangpraseurt et al., 2012a, 2014; Long et al., 2013; Takeshita et al., 2016). For example, the oxygen saturation at the coral-water interface fluctuates from supersaturated during daytime due to production by endosymbionts to hypoxic at night from respiration of the holobiont (Shashar et al., 1993; Kuhl et al., 1995; Gardella and Edmunds, 1999). However, despite these broad chemical changes, oxygen microsensors used in the microenvironment of coral-turf and coral-coralline algae showed that low oxygen concentrations were not generally found at the interface of *Porites* spp., turf algae, and crustose coralline algae (Wangpraseurt et al., 2012a), an observation that illustrated the value of combining microstructure measurements with benthic or flow chamber methods that can take into account multiple reef species.

More microenvironment studies are needed to better understand the metabolic regulation between corals and the surrounding environment (Wangpraseurt et al., 2014). Technological development of microsensors in a modified diver-operated system (Weber et al., 2007) allowed the quantification of irradiance at the coral surface and the measurement of the efficiency of photosynthetic system at polyp and coenosarc microscale (Wangpraseurt et al., 2012b), confirming the ability of corals to adapt to environmental changes, such as temperature or irradiance (Brown et al., 2002). On a broader scale, micro- and automated sensors increasingly have been used in both closed (benthic chamber) and open (reef-scale) approaches to studying coral and reef ecologies. We consider now the subset of unconfined sensor-based studies of whole reef environments.

### Reef Scale Experiments

The study of larger scale reef environments in open or unconfined natural systems was made possible by combining the sensors for measuring both water flow and relevant aspects of water chemistry. A common approach is the DBL method that relies on eddy correlation techniques (Berg et al., 2003), where noninvasive acoustic Doppler velocimeters measuring water flow are coupled with O_2_ sensors to provide three-dimensional fields of oxygen distribution over benthic environments (Long et al., 2013). This method can examine *in situ* dynamics of O_2_ production and consumption (i.e., respiration) in different habitats, including highly productive reef crests relative to reef slopes (Long et al., 2013) as well as cold-water (deep) coral reefs (Rovelli et al., 2015; de Froe et al., 2019). These ecosystem-scale measurements show how high oxygen fluxes are possible, even in nutrient-replete environments such as reef slopes and deep waters. The advantage of such techniques has been discussed by Takeshita et al. (2016) who introduced a new autonomous system (the benthic ecosystem and acidification measurement system) for simultaneous measurement of NCP and NCC from a coral reef through the autonomous use of O_2_ and pH sensing technology. Using this strategy, Takeshita et al. (2018) showed how seawater carbonate chemistry is strongly driven by local benthic metabolism, and that these effects varied substantially over small-scale habitats, across the entire reef platform, and over seasonal timescales, an important perspective for better assessing the impacts of ocean acidification on these ecosystems.

A comparison between the enclosed and open system approaches showed good general agreement with respect to discrete measurements of oxygen from the gradient flux (GF) method at the DBL and a multiparametric probe in a benthic chamber (Yates and Halley, 2003), although other findings indicate that the GF method can offer more accurate measurements of these fluxes (McGillis et al., 2011). The low physical disruption with DBL methods means both less direct impacts on corals and more spatially integrative observation and interpretation of coral reef responses to changes in the natural environment at colony or community levels. Their smaller infrastructure requirements also make them an attractive strategy for investigations. Even so, these approaches can be complementary. For example, DBL studies show that water flow can be a factor affecting the light-limited photosynthesis in coral colonies (Finelli et al., 2006), and more spatially focused studies have used benthic chambers to show that increased water flow leads to increased calcification rates, carbonate deposition, protein concentration, and endosymbionts density (Mass et al., 2011).

Experiments at reef scale represent a key step forward in the *in situ* studies of coral responses in the natural environment (Kline et al., 2012; Albright et al., 2018; Doo et al., 2019; Srednick et al., 2020), but they also have provided the opportunity to quantify reef-scale responses to environmental perturbations. For example, manipulations to lower Ωa by ~20% led to a 34% decrease in NCC consistent with the effect of lower pH on biocalcification (Albright et al., 2016), although this finding may have been influenced by a high abundance of crustose coralline algae living in the reef community (Albright et al., 2018). Another free ocean CO_2_ enrichment (FOCE) system was used to incubate coral reef communities at ambient pCO_2_ (393 μatm) and high pCO_2_ (949 μatm), and a decrease in daily NCC by 49% under high pCO_2_ was observed over a 21-day experiment, corresponding to 26% reduction in NCC per unit of Ωa (Doo et al., 2019). These results were in line with previous studies on ecosystem-level responses of coral reef communities to ocean acidification level projected in the next century. Indeed, a modification of this experimental approach, the coral-proto free ocean carbon enrichment system, allowed the short-term *in situ* study of the induced ocean acidification on coral reef organisms and diel changes of the seawater carbonate system (Kline et al., 2012). Srednick et al. (2020) introduced another novel FOCE for spatial and temporal studies on shallow reefs, with the aim of studying a coral reef community *in situ* under controlled conditions of current and projected levels of pCO_2_. They directly estimated the hysteresis of seawater carbonate chemistry along a reef transect, using high accuracy and precision measurements of seawater pH, pCO_2_, and the biological responses of the reef community with high temporal resolution (Srednick et al., 2020). Other high-frequency sampling of coral reef carbonate dynamics and metabolic rates have used automated systems for measurements of alkalinity, pH, and pCO_2_ at high-frequency sampling (McMahon et al., 2018; Cyronak et al., 2020). Through automated measurements with the slack water and flow respirometry approaches, it is now possible to characterize the net calcification and productivity of a reef system (either reef flats or crests), as well as reveal long-term changes driven by global changes (ocean acidification and global warming) or hysteresis under local changes (Albright et al., 2018; McMahon et al., 2018). However, dissimilarities in the methods used in community metabolism studies (Shaw et al., 2015) create potential uncertainties when applied to coral reefs, in terms of community calcification and seawater carbonate conditions. Limitations of metabolic studies at community level are related to lack of suitable controls, spatial heterogeneities, and thus replicability among sites, as well as confounding factors in open systems, such as the introduction of organisms and differing oceanographic settings in the surrounding reef environments.

## Discussion and Conclusion

We have collated and summarized here the underwater methodologies from 55 studies on coral metabolism and physiology conducted since 1991 (Table 1). The current knowledge of combined effects of local and global stressors comes from a wide breadth of manipulation studies on coral responses under predicted future scenarios but limited in representing complex abiotic factors in ecosystems. Interaction among environmental variables, including temporal changes in inorganic carbon chemistry, physical parameters, or nutrient loads, is an important factor affecting the biogeochemistry of coral health (Doo et al., 2019) and can play a key role in population dynamics. Though invaluable as a study tool, manipulation studies are limited in the ability to mimic important spatial-temporal patterns and interactions. On the other hand, comprehensive long-term *in situ* metabolic measurements still are lacking but nevertheless necessary to understand the energetics and trophodynamics of reef ecosystems.

Laboratory-based studies have provided a strong foundation for understanding coral metabolism and the responses to stress, and they will continue to serve as a primary means of research under controlled conditions. However, the expanding role of *in situ*-based studies of coral systems is essential for extrapolating and modulating these laboratory-based findings to the temporal and spatial complexity of natural reef and environmental conditions. The advantages of *in situ* experimental techniques described here relate to the ability for measuring metabolic and biogeochemical properties of different benthic habitats having both simple (sediments) and complex (corals or rocky bottom) structures. Standardization of methods and replication of experimental studies would allow the simultaneous measures of coral health in different locations with the ability to compare the ecosystem functions. However, there are limitations to *in situ* methodologies. In the case of benthic chambers, there are restrictions to which substrate surfaces are suitable for study, and they are not well suited, even with enhanced flow capabilities, for longer-term monitoring of coral health. These restricted systems also have limited spatial footprints and thus may not adequately account for all benthic of pelagic components (e.g., macroalgae and fish). In the case of DBL approaches, limitations include the needed maintenance and fragility of microsensors, which can limit deployments to shorter duration, or under more quiescent weather conditions.

Long-term continuous observations of coral biological process are critical to the assessment of responses to climate and other anthropogenic drivers and though so far lacking newly developed platforms for general oceanographic study offer this potential moving forward. In particular, autonomous platforms, such as gliders and surface vehicles, are incorporating biogeochemical sensors (i.e., pCO_2_, dissolved oxygen, pH, and chlorophyll a), able to cover spatial, vertical, and temporal observations (Chai et al., 2020; Cryer et al., 2020). While these autonomous devices are not well suited for shallow reef environments, the advances in sensor development and operational constraints will be valuable in designing systems or autonomous robots that can provide reliable long-term assessments of the status of reef ecosystems. Indeed, autonomous measurements of light, dissolved oxygen, and alkalinity were used to estimate the NCP and NCC in order to evaluate the efficacy of coral restoration in supporting the net ecosystem metabolism (Platz et al., 2020). Autonomous sensors can be used also to measure the seasonal variability in carbonate chemistry and the relationship between carbonate chemistry and biological activity of benthic organisms identifying spatial differences according to different substrates, like corals, seagrasses, or mangroves (Meléndez et al., 2020). These systems are especially important in natural ocean acidification laboratories, such as hydrothermal vents, characterized by naturally high fluctuations of pH and temperature (Kerrison et al., 2011; Fabricius et al., 2015; Torres et al., 2021).

Co-deployment of multiple instrumentation approaches, from water chemistry and physics to coral physiology, will be needed to ensure both accuracy and logistic practicality in monitoring fluxes of O_2_, changes of pH, and other aspects of the carbonate system in coral reefs. Even with these accounted for, most studies on coral metabolic rates rely on observation of changes in DIC and alkalinity in a specific (small) area to calculate the carbon fluxes for productivity and biocalcification estimates (Anthony et al., 2011); the movement of seawater flow and aspects of reef heterogeneity cannot be fully taken into account, leading to potentially biased findings that may not adequately reflect natural conditions. Moving forward then, it will be more informative by combining different scales of *in situ* techniques, such as the application of flow respirometry approaches to estimate carbon fluxes over a small spatial scales and different substrate type within a reef, coupled with reef-scale methods that provide an integrative assessment of the temporal variability in coral productivity and growth (McMahon et al., 2018).

## Author Contributions

WD and JC: conceptualization, methodology, formal analysis, and resources. WD: investigation, data curation, and writing – original draft preparation. WD, JC, CC, JW, MW, and LC: writing – review and editing. WD and CC: visualization. LC: supervision. JW and LC: project administration and funding acquisition. All authors contributed to the article and approved the submitted version.

### Conflict of Interest

The authors declare that the research was conducted in the absence of any commercial or financial relationships that could be construed as a potential conflict of interest.
